# Assessment of infection of *Stomoxys calcitrans* larvae by entomopathogenic nematodes *Heterorhabditis amazonensis* NEPT11

**DOI:** 10.29374/2527-2179.bjvm000424

**Published:** 2024-04-05

**Authors:** Américo de Castro Monteiro, Ana Caroline Ferreira de Souza, Danielle Pereira da Silva, Gabriela Pereira Salça de Almeida, Vinícius Teixeira de Souza, João Luiz Lopes Monteiro, Melissa Carvalho Machado do Couto Chambarelli, Avelino José Bittencourt

**Affiliations:** 1 Veterinarian, DSc. Autonomus, Seropédica, RJ, Brazil.; 2 Veterinarian, MSc. Programa de Pós-Graduação em Ciências Veterinárias (PPGCV), Departamento de Parasitologia Animal (DPA). Instituto de Veterinária (IV), Universidade Federal Rural do Rio de Janeiro (UFRRJ). Seropédica, RJ. Brazil.; 3 Undergraduate Student in Veterinary Medicine, IV, UFRRJ. Seropédica, RJ, Brazil.; 4 Agronomist, DSc. Programa de Pós-Graduação em Agronomia (POSAGRO), Departamento de Engenharia Agrícola (DEA). Centro de Ciências Agrárias, Universidade Federal de Roraíma, Cauamé, RR. Brazil.; 5 Veterinarian, DSc. DPA, IV, UFRRJ. Seropédica, RJ. Brazil.; 6 Veterinarian, DSc. Departamento de Medicina e Cirurgia Veterinária (DMCV), IV, UFRRJ. Seropédica, RJ, Brazil.

**Keywords:** NEPT11, biological control, stable fly, NEPT11, controle biológico, mosca-dos-estábulos

## Abstract

This study aimed to evaluate the virulence of *Heterorhabditis amazonenses* NEPT11 against larvae of *Stomoxys calcitrans*. Groups of 10 third-instar fly larvae were deposited in Petri dishes, to which were added 50, 100 and 200 EPNs/larva in 4ml of distilled water. The volume of the control group was the same as the treated group, but without EPNs. Larval mortality was observed daily, until larvae died or adults emerged. The Petri dishes were kept on laboratory shelves at 27 ± 1 °C and 70 ± 10% RH. The experiment was replicated six times. A regression analysis revealed quadratic behavior with increasing concentrations, indicating that the concentration of 200 EPNs/larva (48%) was the most efficient among the tested concentrations, while concentrations of 50 and 100 EPNs/larva killed 26.6 and 40% of larvae, respectively. In general, none of the treatments resulted in a mortality rate of more than 50%, but all the treated groups exhibited a higher mortality than that of the control group. It is concluded that the EPN *H. amazonensis* NEPT11 shows a promising potential to control third-instar larvae of *S. calcitrans*. However, further studies are needed in different situations to better understand the activity of this organism against the immature stages of the stable fly.

## Resumo

Este estudo teve como objetivo avaliar a ação do NEP *Heterorhabditis amazonenses* NEPT11 frente larvas de *S. calcitrans.* Grupos de 10 larvas de terceiro instar da mosca foram depositados em placas de Petri, em seguida, adicionou-se 50, 100, 200, 300 e 400 NEPs/larva em 4ml de água destilada. O volume do grupo controle foi o mesmo dos tratados, porém sem NEPs. A mortalidade das larvas foi observada diariamente, até a morte das larvas ou emergência de adultos. As placas foram mantidas em estantes de laboratório a 27 ± 1 °C e 70 ± 10% UR. O experimento teve seis repetições. Por meio da análise de regressão, foi observado comportamento quadrático com o aumento das concentrações, sendo a concentração de 200 NEPs/larva (48%) a de maior eficiência entre as concentrações testadas, já as concentrações de 50 e 100 NEPs/larva mataram 26,6 e 40% das larvas, respectivamente. De modo geral, nenhum tratamento proporcionou mortalidade superior a 50%, todavia, todos os grupos tratados apresentaram mortalidade superior à observada no controle. Conclui-se que *H. amazonenses* NEPT11 mostrou-se promissor no controle de larvas de terceiro instar de *S. calcitrans*, porém mais estudos devem ser feitos para o melhor entendimento da ação deste organismo frente aos estágios imaturos da mosca-dos-estábulos.

## Introduction

*Stomoxys calcitrans* is a hematophagous dipteran whose life cycle is directly related to the environmental and climatic characteristics of the region where it occurs (Bittencourt, 2012). Its parasitism causes many problems for livestock farming in Brazil, leading to heavy economic losses (Grisi et al., 2014).

The recently reported outbreaks of *S. calcitrans* in Brazil are closely tied to the expansion of the sugarcane agroindustry (Souza et al., 2021). Due to the resistance of pests to chemical pesticides (Barros et al., 2019), allied to the global demand for pesticide-free foods, a variety of microorganisms have been studied to ascertain their possible application in the control of parasites of economic importance.

Entomopathogenic nematodes (EPNs) have emerged as alternatives for use in biological pest control (Kaya & Gaugler, 1993), and these microorganisms have already proved their ability to kill stable fly larvae (Leal et al., 2017; Monteiro Sobrinho et al., 2021), even when the latter are in substrates originating from the sugar and alcohol industry (Monteiro Sobrinho et al., 2016, 2023). Therefore, new species of EPNs must be tested and evaluated in order to increase the alternatives for controlling *S. calcitrans*.

The purpose of this study was to evaluate the virulence of the EPN *Heterorhabditis amazonensis* NEPT11 against third-instar larvae of *S. calcitrans*.

## Material and methods

The *S. calcitrans* colony used in this study was benchtop raised in a laboratory environment (27 ± 1 °C and 70-80% relative humidity – RH), employing an adapted version of the method described by Macedo et al. (2005) and Mello & Garcia (1983).

The EPN colony was raised according to the method described by Lindegren et al. (1993), and it was maintained and multiplied in vivo in *Galleria mellonella* (Lepidoptera: Pyralidae). Infective juveniles (IJs) were placed in a 40mL cell culture flask and stored in an Eletrolab EL 202/4 BOD incubator at 16 ± 1 °C and 70-80% RH for less than 7 days.

To calculate the concentrations used in this study, the IJs were counted in twelve 10μL aliquots taken from an aqueous suspension of EPN. After counting the IJs in the 12 aliquots, the highest and lowest number of EPNs/aliquot were discarded and the average number of IJs in the remaining 10 aliquots was calculated. Based on this calculation, the concentration of suspensions was adjusted to IJs/mL (Taylor et al., 1998).

Groups of 10 third-instar stable fly larvae were placed on Petri dishes (with 2 sheets of filter paper), to which were added 50, 100 and 200 EPNs/larva, diluted in 4ml of distilled water. The volume of water added to the control group was the same as the experimental groups, but without EPNs. Larval mortality was monitored daily, until the death of the larvae or emergence of adult flies. The dishes were kept on laboratory shelves at 27 ± 1 °C and 70 ± 10% RH. The experiment was carried out in a completely randomized design (CRD), with six replications.

The data were subjected to the Shapiro-Wilk normality test and the Bartlett homogeneity of variance test. Once these assumptions were observed, analysis of variance was applied to verify the effects of EPN concentrations, followed by linear regression analysis with the aid of the statistical program SISVAR version 5.1 (Ferreira, 2011).

## Results and discussion

**Figure 1:**
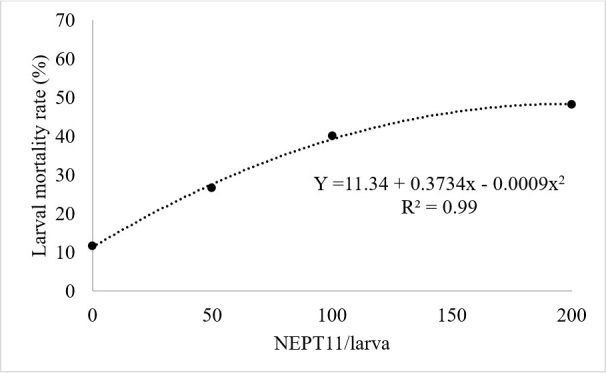
Larval mortality rate of *Stomoxys calcitrans* caused by the EPN *Heterorhabditis amazonensis* NEPT11.

A regression analysis revealed quadratic behavior in response to increasing concentrations, that is, with larval mortality increasing as the concentration of EPNs/larva increased. In which the concentration of 200 NEPs/larva (48%) was the most efficient among the concentrations tested, the concentrations of 50 and 100 NEPs/larva were lower, killing on average 26.6 and 40% of the fly larvae, respectively. In general, no treatment provided mortality greater than 50%, even at the highest concentrations. However, all treated groups presented higher mortality than that observed in the control group (15%) (Figure 1).

A comparison of the results of this study with those of Leal et al. (2017) indicated that the aforementioned authors obtained far superior results than those achieved here. They used a concentration of 200 EPNs/larva of *H. bacteriophora* HP88, which caused a stable fly larvae mortality rate of more than 90%, in contrast to our study, in which the mortality rate at the same concentration did not even reach 50%.

Monteiro Sobrinho et al. (2021), and Leal et al. (2017) reported mortality rates above 90% using *H. bacteriophora* HP88 (200 EPNs/larva) to control *S. calcitrans* larvae. This demonstrates that the nematode *H. bacteriophora* HP88 is considerably more virulent than *H. amazonensis* NEPT11 against stable fly larvae, even when *S. calcitrans* is on other substrates.

Monteiro Sobrinho et al. (2023) reported larvae mortality rates of 73.3% in sugarcane bagasse ash. Studies involving *H. bacteriophora* HP88 and *H. baujardi* LPP7 to control *S. calcitrans* larvae in vinasse, filter cake and sugarcane bagasse (Monteiro Sobrinho et al., 2023) also showed higher mortality rates than those found in the present study, even this not using sugarcane substrate that could interfere in the action of *H. amazonensis* NEPT11. Although the nematode NEPT11 does not cause fly larvae mortality rates above 50%, these results are still superior to those presented in other studies aimed at the biological control of stable fly larvae (Alves et al., 2012; Moraes et al., 2015).

## Conclusions

It is concluded that the EPN *H. amazonensis* NEPT11 showed promise in controlling third-instar larvae of *S. calcitrans*. However, further studies in different situations are needed to better understand the activity of this organism against the immature stages of the stable fly *S. calcitrans*.
